# Improved reproductive outcomes in normogonadotropic oligomenorrheic women undergoing ovarian stimulation with intrauterine insemination: a retrospective cohort analysis of real-world data

**DOI:** 10.3389/fendo.2024.1441796

**Published:** 2024-10-09

**Authors:** Sichen Li, Yao Long, Chunyan Wang, Rui Yang, Junye Lv, Zixin Chen, Jianqiao Liu, Haiying Liu, Mingzhu Cao

**Affiliations:** ^1^ Department of Obstetrics and Gynecology, Center for Reproductive Medicine; Guangdong Provincial Key Laboratory of Major Obstetrics Disease; Guangdong Provincial Clinical Research Center for Obstetrics and Gynecology; Guangdong-Hong Kong-Macao Greater Bay Area Higher Education Joint Laboratory of Maternal-Fetal Medicine; The Third Affiliated Hospital of Guangzhou Medical University, Guangzhou, China; ^2^ Key Laboratory for Reproductive Medicine of Guangdong Province, The Third Affiliated Hospital of Guangzhou Medical University, Guangzhou, China; ^3^ Department of Clinical Medicine, The Third Clinical School of Guangzhou Medical University, Guangzhou, China

**Keywords:** artificial insemination, ovarian stimulation, clinical pregnancy rate, live birth rate, eumenorrheic, oligomenorrheic

## Abstract

**Purpose:**

This study aimed to evaluate the comparative reproductive outcomes of ovarian stimulation combined with intrauterine insemination using partner’s sperm (OS-IUI) in eumenorrheic and normogonadotropic oligomenorrheic women.

**Method:**

A retrospective cohort study was conducted, including 3833 couples who underwent 5920 cycles of OS-IUI between June 2013 and March 2019. Participants were stratified into two cohorts based on menstrual regularity: eumenorrheic and normogonadotropic oligomenorrheic. The primary outcome measured was the live birth rate (LBR) per cycle and cumulative LBR per couple. Secondary outcomes encompassed the clinical pregnancy rate (CPR) per cycle, miscarriage rate, and multiple pregnancy rate. Propensity score matching (PSM) was utilized to balance maternal baseline characteristics.

**Results:**

Prior to PSM, significant differences in CPR, LBR and cumulative LBR were observed between eumenorrheic and oligomenorrheic women, favoring the latter (CPR: 11.16% vs. 18.75%; LBR: 9.02% vs. 14.96%; cumulative LBR: 13.60% vs. 24.25%, *P* < 0.001). These differences persisted post-PSM (CPR: 9.74% vs. 19.29%; LBR: 7.30% vs. 16.29%; cumulative LBR 7.76% vs. 19.90%, *P*<0.001). Multivariate regression analyses revealed that menstrual status was a significant independent predictor of both CPR (adjusted odds ratio [OR]=1.83 before PSM, 2.24 after PSM) and LBR (adjusted OR=1.90 before PSM, 2.46 after PSM). In the subgroup analysis, female age was identified as the sole predictor of reproductive outcomes in oligomenorrheic women undergoing OS-IUI. Conversely, in eumenorrheic women, factors such as age, duration of infertility, body mass index (BMI), ovarian stimulation agents, and the number of dominant follicles were significant influencers of CPR and LBR.

**Conclusion:**

Normogonadotropic oligomenorrheic women demonstrated improved reproductive outcomes with OS-IUI, suggesting that tailored treatment strategies based on menstrual regularity could optimize success rates in infertility management.

## Introduction

Infertility, defined as the inability to achieve a clinical pregnancy after 12 months of regular, unprotected intercourse, is a global health issue affecting an estimated 10-15% of couples worldwide (1). In China, a study by Zheng et al. surveyed 10,742 couples attempting to conceive and reported a prevalence of subfertility as high as 24.9% (2,680 couples) (2). Intrauterine insemination (IUI) with the partner’s sperm is recognized as an initial therapeutic approach for couples facing subfertility (3). IUI is favored for its minimally invasive nature, patient-friendly approach, and cost-effectiveness compared to *in vitro* fertilization (IVF) and intracytoplasmic sperm injection (ICSI) (4). Additionally, IUI has not been associated with an increased risk of pregnancy-related morbidities (5). Despite these advantages, IUI is sometimes undervalued due to its relatively lower reproductive outcomes compared to IVF/ICSI. In China, the reported clinical pregnancy rate (CPR) for IUI is 13.3%, with a delivery rate of 10.5%, while IVF and ICSI have CPRs of 23.2% and 20.5%, and delivery rates of 18.7% and 16.7%, respectively (6). Nonetheless, IUI combined with ovarian stimulation continues to be a preferred first-line treatment for couples with unexplained infertility and mild male factor infertility (7). Given the relatively lower reproductive outcomes of IUI, it is crucial to identify the subfertile population that may benefit more from this treatment modality.

Successful pregnancy requires the interaction of motile sperm and viable oocytes. IUI mimics natural conception by introducing prepared motile sperm into the uterine cavity at the time of ovulation (7). The success of IUI heavily depends on the quality of semen preparation and the timing of ovulation. Ovarian stimulation with intrauterine insemination (OS-IUI) is a commonly employed fertility treatment that aims to enhance the chances of conception in infertile couples. This approach combines ovarian stimulation, which promotes the development of multiple follicles and ovulation, with IUI, which introduces prepared motile sperm directly into the uterine cavity at the time of ovulation. By doing so, OS-IUI seeks to maximize the interaction between viable oocytes and motile sperm, thereby increasing the likelihood of fertilization and pregnancy. Eumenorrheic women, those with regular menstrual cycles, typically exhibit regular ovulatory cycles, and may be expected to respond well to ovarian stimulation, as their bodies are already programmed for regular ovulation. On the other hand, normogonadotropic oligomenorrheic women, who have normal gonadotropin levels but irregular or absent menstrual cycles, may experience sporadic or absent ovulation, and require more intensive ovarian stimulation to achieve optimal follicle development and ovulation (8–11). Ovulatory dysfunction in normogonadotropic oligomenorrheic women can be addressed through ovarian stimulation. The success of OS-IUI in these women may also depend on the precise timing of insemination, as their ovulation patterns are less predictable.

Despite the importance of proper ovulation and the availability of motile sperm, there remains unclear whether eumenorrheic and normogonadotropic oligomenorrheic women can equally benefit from OS-IUI. To address this knowledge gap, the present study compares the reproductive outcomes of eumenorrheic and normogonadotropic oligomenorrheic women following OS-IUI. The comparison between eumenorrheic and normogonadotropic oligomenorrheic women in the context of OS-IUI is crucial to identifying the optimal population for this treatment approach. By understanding how these two groups respond differently to ovarian stimulation and IUI, healthcare providers can tailor fertility treatments more effectively, enhancing the efficacy of OS-IUI and improving reproductive outcomes for infertile couples.

## Methods

### Study population and design

This study was conducted as a retrospective cohort analysis of real-world data of patients who received OS-IUI at our institution between June 2013 and March 2019. The study population comprised 5920 IUI cycles from 3833 couples meeting the following inclusion criteria: 1) females under 40 years of age with at least one patent fallopian tube, as confirmed by hysterosalpingography or laparoscopy; 2) eumenorrheic women with regular menstrual cycle (cycle length ranged from 21 to 35 days), and normogonadotropic oligomenorrheic women. Normogonadotropic women are defined as those with normal levels of gonadotropins (follicle-stimulating hormone (FSH) and luteinizing hormone (LH)) within the reference range for their age and menstrual cycle phase, without other endocrine abnormalities such as hyperprolactinemia or thyroid dysfunction. In the current study, normogonadotropic oligomenorrheic women were identified based on their hormonal profiles and ultrasound findings, confirming oligo-anovulation or anovulation, with menstrual cycle lengths exceeding 35 days; 3) IUI cycles involving ovarian stimulation; 4) presence of 1 to 3 dominant follicles (≥ 14 mm) on the ovulation trigger day; 5) single IUI procedure within a cycle; 6) and no more than 3 cycles of IUI. Exclusion criteria included luteinized unruptured follicles, artificial insemination with donor semen, and incomplete patient information. Indications for IUI included ovulation dysfunction in oligomenorrheic women, mild male factor infertility [defined by sperm concentration ≥ 10 × 10^6^/ml but < 15 × 10^6^/ml and/or progressive motility ≥ 10% but < 32% (12, 13)], unexplained infertility, cervical factor infertility, mild endometriosis, and immunological infertility.

### Ovarian stimulation and IUI procedure

Ovarian stimulation, semen preparation, and insemination procedures were performed according to established protocols (14). Transvaginal ultrasonography was conducted on cycle days 3 to 5 to exclude ovarian cysts larger than 30 mm. All IUI procedure was performed under OS using letrozole (LE), clomiphene citrate (CC), gonadotropins (Gn, including human menopausal gonadotropin, HMG or urine-follitropin, Livzon Corp., China), letrozole and gonadotropins (LE + Gn), or clomiphene citrate and gonadotropins (CC + Gn) based on patient condition, and/or physician and patient preference. The ovarian stimulation protocol was as follows: 1) LE at 2.5-5 mg/d or CC 50-100 mg/d was administered for five consecutive days, starting on cycle days 3 to 5. 2) 37.5-75 IU/d Gn was initiated on cycle days 3 to 5, with varying durations based on the ovarian response. 3) In cases where LE or CC alone did not result in significant follicular recruitment, additional Gn at 37.5 to 112.5 IU/d was provided according to the ovarian response. Follicular development was monitored via transvaginal ultrasound, and hormonal levels (LH, E2, and P) were regularly assessed. Stimulation continued until at least one follicle with a diameter ≥ 17 mm ovulated. Ovulation was triggered with human chorionic gonadotropin (HCG, 6000-10,000 IU, Livzon Corp., China) when the leading follicle reached ≥ 17 mm in diameter, the serum LH level increased, and the leading follicle was at least 14 mm in diameter, or serum progesterone concentrations ≥ 1.5 pg/l with the leading follicle ≥ 14 mm in diameter. A single IUI was performed 12 to 36 hours later. Semen was collected via masturbation and prepared using density gradient centrifugation within one-hour post-ejaculation. Luteal phase support was provided using micronized progesterone (vaginal capsule, 200 mg twice daily), and serum β-HCG levels were assessed approximately 14 days post-insemination.

For cycle management, if the number of dominant follicles on the day of HCG administration exceeded two, cycles were not automatically cancelled. Instead, patients were closely monitored and thoroughly counseled regarding the increased risk of multiple pregnancies associated with multiple dominant follicles. The decision to proceed with or cancel the cycle was made on a case-by-case basis, considering the patient’s preferences and clinical circumstances.

### Outcomes

The primary outcome of this study was the live birth rate (LBR) per cycle and cumulative LBR per couple. Secondary outcomes included CPR, miscarriage rate, and multiple pregnancy rate per cycle. Live birth was determined as the delivery of at least one live offspring after gestational 28 weeks. Cumulative LBR was calculated by dividing the number of women achieving at least one live birth by the total number of women who underwent IUI, considering all cycles until the first live birth was achieved, with a follow-up period of up to two years after the initial OS-IUI cycle. Women were not included in the analysis after having a first delivery. Clinical pregnancy was confirmed by transvaginal ultrasonography 4 to 5 weeks post-insemination. Spontaneous miscarriage was defined as intrauterine pregnancy loss before 27 weeks and 6 days of gestation. Multiple pregnancy was identified by the observation of more than one gestational sac by sonography. All above rates were calculated based on for every insemination cycle but not every initiation cycle (14, 15).

### Statistical analysis

Statistical analyses were conducted using SPSS version 25 (IBM Inc., Armonk, NY, USA). Normal distribution of continuous variables was assessed using the One-Sample Kolmogorov-Smirnov Test. Skewed variables were compared using the Mann-Whitney U test. Categorical variables were expressed as frequencies and proportions and compared using the chi-square test. Multivariate logistic regression analysis was employed to evaluate the independent effects of potential factors on reproductive outcomes. To address potential confounding due to imbalanced baseline characteristics, propensity score matching (PSM) was utilized. The PSM model was developed using logistic regression, and matching was performed without replacement using the nearest neighbor random matching algorithm. The matching ratio was set at 1:1, with factors including female age, duration of infertility, body mass index (BMI), AMH levels, ovarian stimulation agents, and the number of dominant follicles. A caliper value of 0.001 was applied to ensure a precise match while minimizing the reduction in sample size. After matching, 534 cases were paired, and no significant differences in baseline characteristics were observed between the two groups (all *P* > 0.05). Reproductive outcomes for women with regular and irregular menstruation were compared before and after PSM adjustment. All tests were two-sided and *P* value < 0.05 was considered statistically significant.

## Results

### Comparisons of basic clinical characteristics

**Figure 1:**
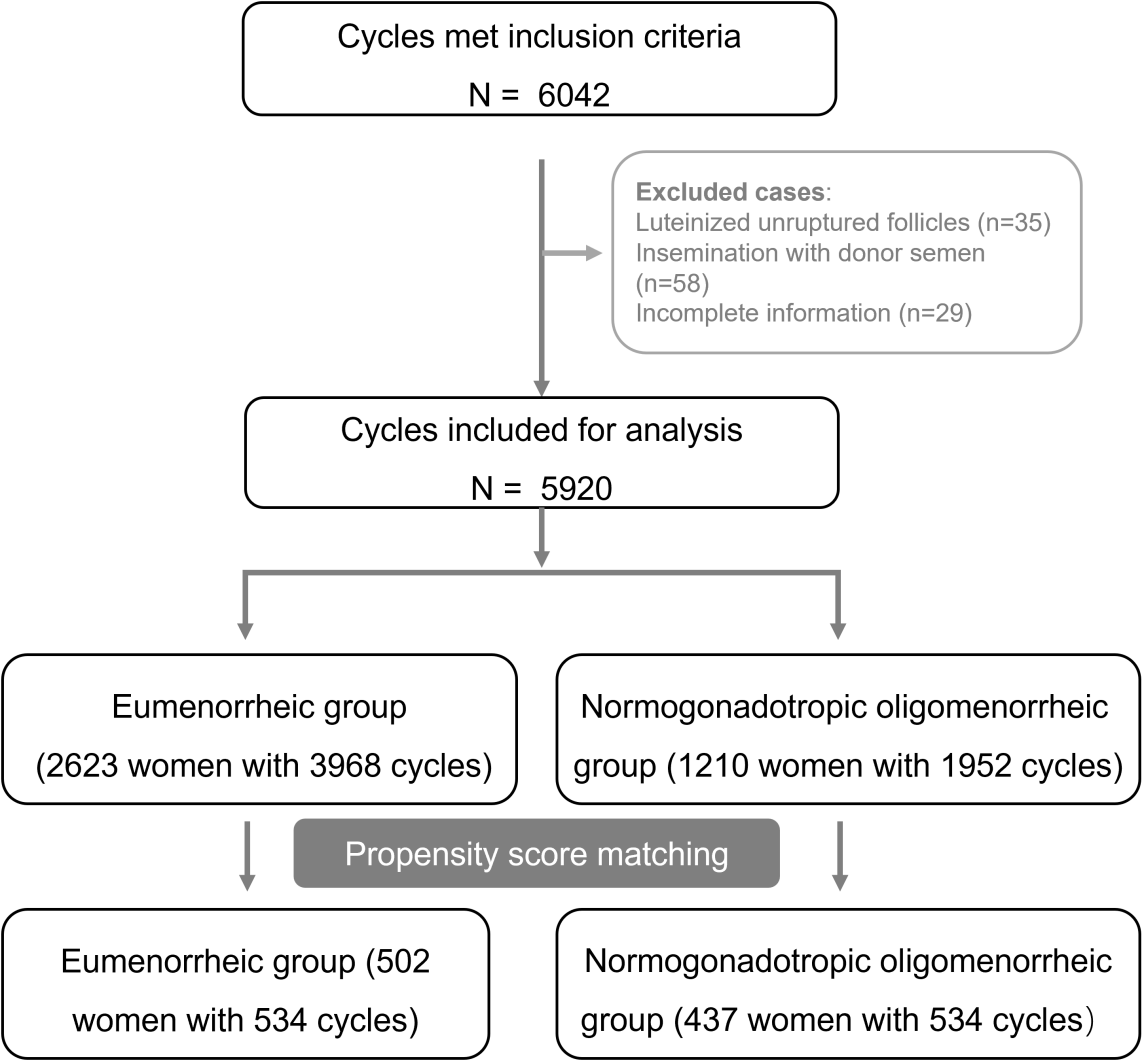
The research design diagram.

Our study encompassed 5920 cycles of OS-IUI, which included 3968 cycles from 2623 eumenorrheic women and 1952 cycles from 1210 oligomenorrheic women. By utilizing PSM algorithm, we successfully paired and assigned 534 cases to both the eumenorrheic and oligomenorrheic groups (shown in **Figure 1**).

The comparative analysis of baseline characteristics, both before and after matching, were presented in **Table 1**. Prior to matching, significant differences were observed between the eumenorrheic and oligomenorrheic groups in terms of maternal age (median 31 vs. 30 years old, *P* < 0.001), duration of infertility (3.47 ± 2.08 vs. 3.70 ± 2.20 years, *P* = 0.002), BMI (21.37 ± 3.15 vs. 22.24 ± 5.93 kg/m^2^, *P* < 0.001), methods of ovarian stimulation (*P* < 0.001), and endometrial thickness (8.43 ± 2.67 vs. 8.60 ± 2.58 mm, *P* = 0.046). Following PSM, no significant differences were detected in the basic clinical characteristics between the eumenorrheic and oligomenorrheic groups, indicating a successful balance of the cohorts. This matching process ensured that subsequent analyses were conducted on comparable groups, thereby enhancing the validity of the study findings.

**Table 1:** Basic clinical characteristics and fertility treatment results of OS-IUI.

	Before PSM			After PSM		
Characteristics	Eumenorrheic	Oligomenorrheic	*P*	Eumenorrheic	Oligomenorrheic	*P*
	N = 3968	N = 1952		N = 534	N = 534	
Female age (years old)	31 (26, 36)	30 (26, 34)	< 0.001	31 (26, 36)	30 (25, 35)	0.472
Duration of infertility (years)	3.47 ± 2.08	3.70 ± 2.20	0.002	3.54 ± 1.82	3.50 ± 2.11	0.056
Female BMI (kg/m^2^)	21.37 ± 3.15	22.24 ± 5.93	< 0.001	21.49 ± 3.18	21.44 ± 3.29	0.951
AMH (ng/ml)	4.80 ± 3.43	8.04 ± 4.56	< 0.001	5.77 ± 3.54	5.80 ± 3.48	0.606
Basal FSH(u/L)	5.57 ± 1.41	5.20 ± 1.33	< 0.001	5.42 ± 1.45	5.25 ± 1.48	0.052
Basal LH(u/L)	3.78 ± 1.88	5.75 ± 4.07	< 0.001	3.70 ± 1.71	4.72 ± 3.10	0.001
Basal E_2_(pmol/L)	128.01 ± 48.28	122.11 ± 47.54	< 0.001	126.19 ± 58.57	119.65 ± 48.44	0.065
Ovulation stimulating (n/%)			< 0.001			0.907
LE	402/10.43%	164/8.56%		51/9.55%	52/9.74%	
LE + Gn	120/ 3.11%	172/8.98%		23/4.31%	21/3.93%	
CC	448/11.62%	201/10.50%		38/7.12%	46/8.61%	
CC + Gn	132/3.42%	232/12.11%		17/3.18%	15/2.81%	
Gn	2753/71.41%	1146/59.84%		405/75.84%	400/74.90%	
Type of infertility (n/%)			0.111			0.296
Primary infertility	2547/64.22%	1295/ 66.34%		368/68.91%	352/65.92%	
Secondary infertility	1419/35.78%	657/33.66%		166/31.09%	182/34.08%	
Couples with mild male factor (n/%)			0.669			0.426
Mild male factor	1926/48.54%	959/49.13%		283/53.00%	270/50.56%	
Indications for IUI (n/%)			< 0.001			0.205
Unexplained infertility	1718 /43.30%	918/47.03%		211/39.51%	237/44.38%	
Cervical infertility	56/1.41%	21/1.08%		3/0.56%	3/0.56%	
Mild male factor	1926/48.54%	959/49.13%		283/53.00%	270/50.56%	
Mild endometriosis	268/6.75%	54/2.77%		37/6.93%	24/4.49%	
TMSC after sperm preparation (×10^6^)	29.27 ± 25.05	30.21 ± 25.45	0.104	27.43 ± 22.57	28.43 ± 24.85	0.054
Hormonal levels on HCG day						
E_2_(pmol/L)	1586.12 ± 835.15	1618.32 ± 850.16	0.234	1583.80 ± 788.36	1532.90 ± 798.69	0.340
LH(u/L)	15.91 ± 13.35	13.82 ± 12.17	< 0.001	14.62 ± 12.07	13.79 ± 12.29	0.326
P(nmol/L)	1.56 ± 1.32	1.36 ± 1.25	< 0.001	1.40 ± 1.10	1.27 ± 1.08	0.109
Number of follicles ≥ 14 mm on HCG day (n/%)			0.834			0.844
One	3062/77.17%	1493/76.49%		402/75.28%	410/76.78%	
Two	745/18.78%	376/19.26%		109/20.41%	103/19.29%	
Three	161/4.06%	83/4.25%		23/4.31%	21/3.93%	
Endometrial thickness on HCG day (mm)	8.43 ± 2.67	8.60 ± 2.58	0.046	8.62 ± 2.50	8.75 ± 2.48	0.383
Cancellation due to multi-follicle %(n)	2.48% (101/4069)	5.43% (112/2064)		NA	NA	

IUI, intrauterine insemination with husband sperm; PSM, propensity score matching; BMI, body mass index; AMH, anti-Mullerian hormone; LE, letrozole; CC, clomiphene citrate; Gn, gonadotropin; TMSC, total motility sperm count; HCG, human chorion gonadotropin.

The overall cancellation rate due to multiple follicular development was 2.48% (101/4069) in eumenorrheic women and 5.43% (112/2064) in normogonadotropic oligomenorrheic women. These cancellation rates refer to cycles that were cancelled prior to IUI and are not included in the analyzed data, which focuses solely on cycles where IUI was performed. Therefore, the presented results represent the outcomes of treatment cycles that proceeded to IUI. After PSM, only cycles with IUI performed were matched, ensuring that the comparison of outcomes was based on comparable groups. Consequently, no cancelled cycles were included in the PSM analysis.

### Comparisons of reproductive outcomes of OS-IUI between eumenorrheic and oligomenorrheic group

The analysis of reproductive outcomes following OS-IUI procedures revealed significant differences between eumenorrheic and oligomenorrheic women, as detailed in **Table 2**. Before the PSM, the CPR was markedly higher in the oligomenorrheic group compared to the eumenorrheic group, with percentages of 18.75% (366/1952) versus 11.16% (443/3968), respectively (*P* < 0.001). Similarly, the LBR was also higher in the oligomenorrheic group, at 14.96% (292/1952) compared to 9.02% (358/3968) in the eumenorrheic group (*P* < 0.001). A similar trend was also observed considering of cumulative LBR (13.65% vs. 24.13%, *P* < 0.001). After PSM, these differences persisted, with the oligomenorrheic group maintaining a higher CPR of 19.29% (103/534) compared to the eumenorrheic group’s 9.74% (52/534) (*P* < 0.001). Likewise, the LBR per cycle and cumulative LBR per couple remained significantly higher in the oligomenorrheic group compare with eumenorrheic group (LBR per cycle: 7.30% vs. 16.29%, and cumulative LBR per couple 7.76% vs. 19.90%, *P* < 0.001). These results suggest that, after adjusting for confounding factors, oligomenorrheic women undergoing OS-IUI have a better chance of achieving a clinical pregnancy and live birth per cycle, and this benefit was also evident in cumulative live birth. Nevertheless, there were no significant differences in the rates of spontaneous miscarriage, ectopic pregnancy, and multiple pregnancy between the two groups, both before and after PSM.

**Table 2:** Reproductive outcomes of OS-IUI between cycles with eumenorrheic and oligomenorrheic women.

Reproductive outcomes %(n)	Before PSM			After PSM		
	Eumenorrheic	Oligomenorrheic	*P*	Eumenorrheic	Oligomenorrheic	*P*
	N = 3968	N = 1952		N = 534	N = 534	
Clinical pregnancy rate	11.16% (443/3968)	18.75% (366/1952)	< 0.001	9.74% (52/534)	19.29% (103/534)	< 0.001
Live birth rate	9.02% (358/3968)	14.96% (292/1952)	< 0.001	7.30% (39/534)	16.29% (87/543)	< 0.001
Cumulative live birth rate	13.60% (357/2625)	24.25% (293/1208)	< 0.001	7.76% (39/502)	19.90% (87/437)	< 0.001
Spontaneous miscarriage rate	16.82% (73/434)	18.11% (65/359)	0.635	22.00% (11/50)	15.53% (16/103)	0.325
Ectopic pregnancy rate	2.48% (11/443)	1.91% (7/366)	0.584	3.85% (2/52)	0.00% (0/103)	0.111
Multiple pregnancy rate	6.09% (27/443)	9.02% (33/366)	0.114	7.69% (4/52)	6.80% (7/103)	0.837

IUI, intrauterine insemination with husband sperm; PSM, propensity score matching.

### The effect of menstruation status on reproductive outcomes

To evaluate the influence of menstrual status on CPR and LBR per cycle, we conducted univariate and multivariate regression analyses both before and after PSM. The results, as detailed in **Table 3**, consistently indicated a significant association between oligomenorrheic status and improved reproductive outcomes. Before PSM, univariate regression analysis revealed that oligomenorrheic women had a higher likelihood of achieving a clinical pregnancy (OR=1.84, 95% CI=1.58-2.13; *P* < 0.001) and live birth (OR=1.77, 95% CI=1.50-2.09; *P* < 0.001). After adjusting for potential confounders in the multivariate regression model, the association remained robust, with adjusted ORs of 1.83 (95% CI =1.52-2.21) for CPR and 1.90 (95% CI =1.54-2.34) for LBR, again both statistically significant (*P <*0.001). Post-PSM, the univariate analysis continued to demonstrate a significant increase in the likelihood of clinical pregnancy (OR=2.22, 95% CI=1.55-3.17) and live birth (OR=2.47, 95%=CI 1.66-3.68) in oligomenorrheic women. The multivariate regression analysis, which accounted for baseline characteristics, further confirmed these findings with adjusted ORs of 2.24 (95% CI 1.44-3.49) for CPR and 2.46 (95% CI = 1.50-4.02) for LBR, both indicating a higher chance of these outcomes in oligomenorrheic women (*P* < 0.001). The consistency of these results across both univariate and multivariate analyses, and their persistence after PSM, underscored the independent effect of menstrual status on reproductive outcomes following OS-IUI.

**Table 3:** Univariate and multivariate regression analyses of menstruation status on reproductive outcomes.

	Univariate regression OR (95% CI)			Multivariate regression Adjusted OR (95% CI)		
	Eumenorrheic	Oligomenorrheic	*P*	Eumenorrheic	Oligomenorrheic	*P*
Before PSM						
Clinical pregnancy	Ref	1.84 (1.58 - 2.13)	< 0.001	Ref	1.83 (1.52 - 2.21)	< 0.001
Live birth	Ref	1.77 (1.50 - 2.09)	< 0.001	Ref	1.90 (1.54 - 2.34)	< 0.001
After PSM						
Clinical pregnancy	Ref	2.22 (1.55 - 3.17)	< 0.001	Ref	2.24 (1.44 - 3.49)	< 0.001
Live birth	Ref	2.47 (1.66 - 3.68)	< 0.001	Ref	2.46 (1.50 - 4.02)	< 0.001

OR, odds ratio; 95% CI, 95% confidential index; PSM, propensity score matching; Ref, reference.

### Factors affecting the reproductive outcomes of OS-IUI within eumenorrheic and oligomenorrheic group

To discern the determinants influencing the reproductive outcomes of OS-IUI within regular and irregular menstrual groups, we performed multivariate logistic regression analyses. The findings, as outlined in **Tables 4**, **5**, shed light on several factors with independent impacts on CPR and LBR for each menstrual group. In the eumenorrheic group, female age and the duration of infertility were identified as negative predictors for both CPR and LBR, with adjusted ORs of 0.97 (95% CI 0.93-0.99) and 0.90 (95% CI 0.85-0.97) for CPR, and 0.95 (95% CI 0.92-0.99) and 0.88 (95% CI 0.82-0.96) for LBR, respectively. Conversely, female BMI demonstrated a positive association with both CPR and LBR, with adjusted ORs of 1.05 (95% CI 1.00-1.09 P = 0.021) and 1.06 (95% CI 1.01-1.10; *P* = 0.009), respectively. The TMSC after sperm preparation was another factor positively associated with reproductive outcomes, with adjusted ORs of 1.01 (95% CI 1.00-1.01; *P*<0.01) for both CPR and LBR, indicating that higher BMI and TMSC were linked to better reproductive outcomes. Furthermore, the type of ovarian stimulation used had a significant impact. Specifically, cycles induced with clomiphene citrate and gonadotropins (CC + Gn) or gonadotropins alone (Gn) exhibited numerically higher CPR and LBR compared to those induced with letrozole (LE). The adjusted ORs for CPR were 2.43 (95% CI 1.2-4.86; *P* = 0.012) for CC + Gn and 1.92 (95% CI 1.20-3.06; *P* = 0.006) for Gn, while for LBR, the adjusted ORs were 3.29 (95% CI 1.53-7.05; *P* = 0.002) for CC + Gn and 2.11 (95% CI 1.21-3.68; *P* = 0.008) for Gn. Additionally, a higher number of dominant follicles on HCG day was predictive of increased CPR and LBR, as evidenced by adjusted ORs of 1.51 (95% CI 1.13-2.02; *P* = 0.006) for two follicles and 1.70 (95% CI 1.02-2.85; *P* = 0.042) for three follicles in CPR, as well as 1.54 (95% CI 1.11-2.13; *P* = 0.010) for two follicles in LBR.

**Table 4:** Multivariate logistic regression analyses for clinical pregnancy and live birth outcomes within cycles of eumenorrheic women.

	Cycles of eumenorrheic women (N=3968)			
	Clinical pregnancy Adjusted OR (95% CI)	*P*	Live birth Adjusted OR (95% CI)	*P*
Female age (years old)	0.97 (0.93 -0.99)	0.049	0.95 (0.92 - 0.99)	0.017
Duration of infertility (years)	0.90 (0.85 - 0.97)	0.003	0.88 (0.82 - 0.96)	0.002
Female BMI (kg/m^2^)	1.05 (1.00 - 1.09)	0.021	1.06 (1.01 - 1.10)	0.009
AMH (ng/ml)	1.00 (0.99 - 1.00)	0.804	1.00 (0.99 - 1.01)	0.844
Cycle No.	0.88 (0.73 - 1.05)	0.155	0.88 (0.72 - 1.08)	0.229
Type of infertility				
Primary	Ref		Ref	
Secondary	1.09 (0.84 - 1.41)	0.516	1.07 (0.80 - 1.43)	0.652
Ovulation stimulating (%)				
LE	Ref		Ref	
LE + Gn	1.65 (0.75 - 3.65)	0.213	1.95 (0.79 - 4.78)	0.145
CC	1.23 (0.69 - 2.20)	0.482	1.45 (0.74 - 2.84)	0.275
CC + Gn	2.43 (1.21 - 4.86)	0.012	3.29 (1.53 - 7.05)	0.002
Gn	1.92 (1.20 - 3.06)	0.006	2.11 (1.21 - 3.68)	0.008
Infertile factors				
Unexplained	Ref		Ref	
Cervical	0.65 (0.15 - 2.82)	0.561	0.86 (0.20 - 3.81)	0.845
Mild male factor	1.03 (0.80 - 1.32)	0.835	0.97 (0.74 - 1.29)	0.855
Mild endometriosis	0.92 (0.55 - 1.55)	0.758	0.92 (0.51 - 1.64)	0.768
TMSC after sperm preparation (×10^6^)	1.01 (1.001 - 1.01)	0.001	1.01 (1.001 - 1.01)	0.004
Number of follicles ≥ 14 mm on HCG day				
One	Ref		Ref	
Two	1.51 (1.13 – 2.02)	0.006	1.54 (1.11 - 2.13)	0.010
Three	1.70 (1.02 - 2.85)	0.042	1.72 (0.97 - 3.03)	0.061
Endometrial thickness on HCG day (mm)	1.00 (0.96 - 1.05)	0.945	0.99 (0.94 - 1.04)	0.755

IUI, intrauterine insemination with husband sperm; OR, odds ratio; 95% CI, 95% confidential index; BMI, body mass index; AMH, anti-Mullerian hormone; LE, letrozole; CC, clomiphene citrate; Gn, gonadotropin; TMSC, total motility sperm count; HCG, human chorion gonadotropin.

**Table 5:** Multivariate logistic regression analyses for clinical pregnancy and live birth outcomes within cycles of oligomenorrheic women.

	Cycles of oligomenorrheic women (N=1952)			
	Clinical pregnancy OR (95% CI)	*P*	Live birth OR (95% CI)	*P*
Female age (years old)	0.96 (0.92 - 1.00)	0.068	0.95 (0.91 - 0.99)	0.031
Duration of infertility (years)	0.94 (0.88 - 1.01)	0.115	0.93 (0.86 - 1.01)	0.074
Female BMI (kg/m^2^)	1.01 (0.99 - 1.03)	0.486	1.01 (0.99 - 1.01)	0.459
AMH (ng/ml)	1.01 (0.98 - 1.04)	0.621	1.00 (0.99 - 1.03)	0.927
Cycle No.	1.11 (0.89 - 1.37)	0.355	1.02 (0.80 - 1.28)	0.898
Type of infertility				
Primary	Ref		Ref	
Secondary	1.03 (0.76 - 1.40)	0.837	1.07 (0.77 - 1.49)	0.675
Ovulation stimulating (%)				
LE	Ref		Ref	
LE + Gn	1.22 (0.66 - 2.26)	0.534	1.65 (0.82 - 3.31)	0.158
CC	0.64 (0.32 - 1.25)	0.188	0.91 (0.43 - 1.91)	0.800
CC + Gn	0.92 (0.51 - 1.69)	0.799	1.21 (0.61 - 2.41)	0.581
Gn	1.04 (0.63 - 1.69)	0.888	1.41 (0.80 - 2.48)	0.237
Infertile factors				
Unexplained	Ref		Ref	
Cervical	1.01 (1.48 - 21.91)	0.011	2.84 (0.69 - 11.74)	0.149
Mild male factor	1.02 (0.76 - 1.36)	0.902	1.03 (0.76 - 1.41)	0.830
Mild endometriosis	0.44 (0.13 - 1.47)	0.180	0.56 (0.17 - 1.88)	0.346
TMSC after sperm preparation (×10^6^)	1.00 (0.99 -1.01)	0.686	1.00 (0.99 - 1.01)	0.942
Number of follicles ≥ 14 mm on HCG day				
One	Ref		Ref	
Two	1.27 (0.91 - 1.78)	0.165	1.28 (0.89 - 1.83)	0.185
Three	1.38 (0.75 - 2.55)	0.297	1.05 (0.52 - 2.12)	0.895
Endometrial thickness on HCG day (mm)	1.05 (0.99 - 1.11)	0.102	1.03 (0.97 - 1.10)	0.312

IUI, intrauterine insemination with husband sperm; OR, odds ratio; 95% CI, 95% confidential index; BMI, body mass index; AMH, anti-Mullerian hormone; LE, letrozole; CC, clomiphene citrate; Gn, gonadotropin; TMAC, total motility sperm count; HCG, human chorion gonadotropin.

In the oligomenorrheic group, advanced female age was associated with a decreased LBR, with an adjusted OR of 0.95 (95% CI 0.91-0.99; *P*=0.031), suggesting a lower likelihood of live birth with increasing age. Women with cervical factors had an increased CPR (adjusted OR = 1.01, 95% CI 1.48-21.91; *P* = 0.011), although the association did not reach conventional levels of statistical significance in LBR (adjusted OR = 2.84, 95% CI 0.69-11.74; *P* = 0.149). The impact of female BMI, AMH levels, and cycle number on reproductive outcomes was not statistically significant in the oligomenorrheic group, indicating that these factors may be less influential in this population.

## Discussion

The present study’s findings, derived from a comprehensive retrospective analysis of 5920 OS-IUI cycles, contribute significantly to the field of reproductive medicine. Our results indicated that oligomenorrheic women experienced superior CPR and LBR per cycle as well as cumulative LBR per couple following OS-IUI when compared to eumenorrheic women, both before and after PSM. This observation holds substantial clinical relevance, as it suggests that OS-IUI may be a particularly effective treatment option for women with menstrual irregularities, a common characteristic in those with infertility.

Successful conception relies on various factors, including adequate motile sperm count, ovulation, and fallopian tube patency. OS-IUI has demonstrated efficacy in treating infertility due to unexplained or mild male factors (16). The use of ovarian stimulation agent may rectify subtle ovulatory disorder (1, 17). Additionally, IUI bypasses the cervical barrier, allowing for a more direct placement of prepared, highly motile sperm into the uterine cavity. This not only ensures that a higher concentration of sperm reaches the fallopian tubes but also reduces the risk of sperm being hindered by cervical mucus abnormalities or other factors that might impede their progress. The efficacy of OS-IUI in promoting fertility can be attributed to the combined effect of these two interventions: ovarian stimulation, which provides one or more optimal follicles, and IUI, which ensures a sufficient number of motile sperm are available in the uterus at the optimal time for fertilization. This synergy increases the likelihood of successful conception by maximizing the chances of sperm and egg encountering each other under favorable conditions (16, 18). Mechanistically, ovarian stimulation primes the ovaries to produce follicles containing mature eggs, while IUI ensures that sperm are efficiently transported past potential barriers and concentrated in the vicinity of the fallopian tubes. The resulting increase in the number and quality of available gametes, as well as the timing of their encounter, enhances the chances of fertilization and subsequent implantation, leading to improved pregnancy outcomes both per cycle and cumulatively over time. Importantly, even when controlling for baseline characteristics and cycle features, the cumulative benefit of OS-IUI in terms of live birth rates persists, highlighting its effectiveness as a fertility treatment option.

When compared to IVF, OS-IUI presents as a cost-effective and less invasive option for couples with unexplained infertility, mild male factors, cervical factors, and mild endometriosis (18). However, the primary criticism of IUI is its lower success rate compared to IVF. The 2013 guidelines from the UK National Institute for Health and Care Excellence (NICE) recommended against the use of IUI for couples with unexplained infertility, mild endometriosis, and mild male factors (19). Despite of this, IUI continues to be offered in many institutions due to its aforementioned benefits. In China, OS-IUI remains as the first-line treatment for unexplained infertile couples and those with mild male factors (20). A publication supporting the NICE guidelines suggested that ovarian stimulation with IUI or IUI in a natural cycle does not significantly improve live birth outcomes compared to expectant management (21). However, the choice of treatment may not be the sole factor influencing this conclusion; rather, the selection of an appropriate patient population may be the key influencer. Women with specific characteristics may benefit more from OS-IUI than the general population. The primary objective of this study is to identify the optimal population for OS-IUI. Specifically, we aim to determine whether eumenorrheic women, who have normal ovulation, or oligomenorrheic women, who achieve ovulation with the aid of ovarian stimulation, can benefit from OS-IUI with improved reproductive outcomes.

Our study revealed a significant improvement in successful pregnancy rates among women with oligomenorrhea. This enhancement in reproductive outcomes persisted in oligomenorrheic women even after adjusting for confounding factors such as female age, infertility duration, and ovarian reserve tests, aligning them with their eumenorrheic counterparts. A compelling rationale underpinning this phenomenon is that ovulation stimulation (OS) precisely tackles the core infertility challenge in oligomenorrheic individuals, often manifested as anovulation or suboptimal ovulation. By optimizing the ovulation process, OS, when coupled with intrauterine insemination (IUI), significantly boosts the chances of conception.

A plausible rationale for this observation is that OS directly tackles their primary infertility challenge, in oligomenorrheic individuals, often manifested as anovulation or suboptimal ovulation. By optimizing the ovulatory process, OS, when coupled with IUI, boosts the likelihood of achieving pregnancy. In contrast, eumenorrheic women, In contrast, eumenorrheic women, whose menstrual cycles are typically regular and accompanied by adequate ovulation, may encounter infertility barriers rooted in different etiologies. These may encompass fertilization impediments despite sperm motility within normal ranges or instances of oocyte maturation arrest, which are less directly addressed by OS-IUI protocols. (22–24). Consequently, the therapeutic benefit of OS-IUI may be mitigated in this population, as it does not comprehensively target the underlying causes of their infertility. Moreover, the presence of subtle endometrial abnormalities or receptor deficiencies in oligomenorrheic patients, which may be undetected or underappreciated, could also contribute to their improved response to OS-IUI. Enhanced endometrial receptivity following ovulation induction may create a more conducive environment for embryo implantation, thus facilitating pregnancy. Conversely, in eumenorrheic women, the endometrial environment may already be optimized, limiting the additional benefit conferred by ovulation stimulation. This aligns with findings from other studies, that suggested OS-IUI may not significantly enhance reproductive outcomes in women without ovulatory disorders (25, 26). Devilbiss et al. also reported that sporadic anovulation in regularly menstruating women had a minimal effect on cumulative pregnancy rates, which may explain why OS-IUI does not substantially improve outcomes in eumenorrheic women compared to oligomenorrheic ones (27).

The decision to proceed with IUI should be highly individualized, considering the couple’s specific circumstances. Identifying factors that influence the success of OS-IUI is crucial prior to medical consultation. Our study aimed to delineate key determinants of pregnancy to maximize the probability of conception following OS-IUI. The significance of maternal age is underscored by its correlation with fertility decline; thus, IUI may offer limited benefits to women over the age of 40 (28, 29). Our cohort included only couples under 40 years of age, undergoing up to three IUI cycles. This approach aligns with other studies that reported a decrease in pregnancy rates with increasing maternal age (30–32). OS-IUI, as a primary treatment option, allows infertile women with irregular menstrual cycles to potentially bypass unnecessary IVF procedures, especially younger couples. Nonetheless, caution is advised when recommending OS-IUI to older women.

In addition to maternal age, the duration of infertility and BMI were also pivotal before proceeding with OS-IUI for eumenorrheic women. Both factors are widely acknowledged as vital predictors of pregnancy outcomes (33). The positive association between BMI and reproductive success in eumenorrheic women suggests that optimal weight management may be conducive to enhance the efficacy of fertility treatments. Notably, when infertility extends beyond six years, the success rate of IUI diminishes significantly (34). The lower pregnancy rates associated with OS-IUI could result in an extended time to conception, impacting fertility, particularly in women with advanced age and prolonged infertility. Consequently, OS-IUI may be more advantageous for younger subfertile eumenorrheic women with a shorter history of subfertility (35). Recommending OS-IUI to older eumenorrheic women with a longer duration of subfertility should be done with prudence.

Bi-follicular stimulation has been reported to enhance the chances of pregnancy in subfertile women with regular menstruation (14, 36). Our IUI protocol stipulated that the number of dominant follicles (with diameters ≥ 14mm) should not exceed three to minimize the risk of multiple pregnancies (37). Achieving the presence of two dominant follicles is considered an optimal goal for ovarian stimulation in IUI, balancing pregnancy chances with the risk of multiples (38). The type of ovarian stimulation used also emerged as a significant factor. Our results indicate that CC + Gn or Gn-only stimulation protocols led to better outcomes compared to LE-induced cycles. This could be due to the fact that gonadotropins promote a more robust follicular development and are associated with higher pregnancy rates (39). These findings are supported by other studies that have shown the efficacy of gonadotropin-based stimulation in IUI cycles (40, 41).

To the best of our knowledge, this study is one of the pioneering efforts to elucidate the influence of menstrual regularity on the pregnancy outcomes of OS-IUI. By leveraging a substantial sample size and a comprehensive statistical approach, including multivariable regression analysis and PSM processing, we minimized the impact of potential confounders and established a robust correlation between menstrual regularity and the success rate of OS-IUI. Our study provides a detailed comparison of reproductive outcomes between eumenorrheic and normogonadotropic oligomenorrheic women undergoing OS-IUI. While it is well-established that women with ovulation disorders, such as oligomenorrhea, benefit from ovulation induction, our study offers new insights by directly comparing their outcomes with those of eumenorrheic women, who have regular menstrual cycles but may still experience infertility. The primary significance of our study lies in its focus on the differential reproductive outcomes between these two distinct groups. By emphasizing the importance of personalized treatment strategies based on menstrual regularity, our findings suggest that normogonadotropic oligomenorrheic women may achieve better reproductive outcomes with OS-IUI compared to eumenorrheic women. This information can guide clinicians in making informed decisions about treatment options for their patients. Additionally, our study utilizes a large dataset of real-world clinical data, providing robust evidence on the effectiveness of OS-IUI in different subgroups of infertile women. The use of PSM to adjust for potential confounders further enhances the reliability of our findings and supports the validity of our conclusions. Nonetheless, our study was not without limitations. Certain variables, such as the number of antral follicles, were not included in the analysis due to the retrospective nature of the study. Because this study was based on retrospective analysis of real-world data, cumulative LBR was determined on actual number of finished cycles rather than predetermined number of cycles until they reach successful pregnancy. The dropout of participants who failed the first or second cycle cannot be completely avoided. A well-designed prospective study is warranted to validate our findings.

In conclusion, our study indicated that infertile oligomenorrheic women experience more favorable pregnancy outcomes following OS-IUI compared to their eumenorrheic counterparts. The implementation of OS-IUI strategies should be considerate of menstrual status. Young couples dealing with oligomenorrhea should be particularly encouraged to pursue OS-IUI. Similarly, for young eumenorrheic women with a brief history of infertility, OS-IUI is a viable recommendation. Employing CC plus Gn or Gn alone for ovarian stimulation and aiming for bi-follicular development may lead to enhanced reproductive outcomes. Consequently, it is judicious to prioritize OS-IUI as a treatment option for normogonadotropic oligomenorrheic women facing infertility challenges.

## Data Availability

The raw data supporting the conclusions of this article will be made available by the authors, without undue reservation.
